# Editorial: Fungi as cell factories: Genetic engineering and applications

**DOI:** 10.3389/fbioe.2022.1109992

**Published:** 2022-12-14

**Authors:** Sonja Billerbeck, Anderson G. Oliveira, A. Pedro Gonçalves

**Affiliations:** ^1^ Molecular Microbiology, Groningen Biomolecular Sciences and Biotechnology Institute, University of Groningen, Groningen, Netherlands; ^2^ Department of Chemistry and Biochemistry, Yeshiva University, New York, NY, United States; ^3^ College of Medicine, National Cheng Kung University, Tainan, Taiwan

**Keywords:** genetic engineering, fungal biotechnology, synthetic biology, secretion, fermentation, biosynthesis, cell factory

Wherever they are found—on nearly any substrate on Earth—fungal organisms have a key role in promoting the homeostasis of the ecosystems. They are important decomposers in the soil food web due to their role in converting organic matter that is hard to digest into forms that other organisms can use. Fungi are not only beneficial for natural processes but have also become crucial players in engineered bioprocesses within the new bioeconomy due to their functionally diverse capacities. Fungi encompass a large group of organisms, ranging from well-studied species (*e.g.*, *Saccharomyces cerevisiae*) to more complex filamentous fungi, including mushroom-forming species. Several fungal species have shown amenability to genetic engineering and large-scale production, but many are yet to be explored.

Fungal biotechnology has come a long way in the last couple of decades. In this Research Topic we consolidated some new developments in: 1) creating tools for cell-factory engineering (Mózsik et al. and Baeza et al.); 2) actual examples of how fungi can be turned into cell factories for the production of valuable chemicals (Wei et al., Ruger-Herreros et al. and Zhao et al.) or environmental monitoring (Soares et al.).

The review by Mózsik et al. laid out a comprehensive overview of available strategies for the transcriptional activation of often silent secondary metabolites-encoding gene clusters in filamentous fungi. Those include perturbing chromatin-based regulation, activating genes by overexpressing global or cluster-specific regulators, or via orthogonal tools such as synthetic zinc-finger-based transcription factors or CRISPR activation. Being able to activate these clusters is a straightforward means to turn the natural producer into a cell factory for relevant secondary metabolites. This can have advantages over bringing a gene cluster into a heterologous host: the biosynthetic enzymes are likely functional, relevant precursors are likely available and the host is likely not intoxicated by the product.

Baeza et al. delivered a study that optimizes a previously developed blue-light-switchable transcription activation system. The authors employed the FUN-LOV (FUNgal Light Oxygen and Voltage) optogenetic switch, drug resistance markers, and a luciferase-based reporter system to prove the feasibility of this strategy to fine-tune gene expression for usage in industrial and wine yeast. Tools for light-based activation of gene expression are industrially relevant, as light is cheap in comparison to often expensive chemical inducers that are currently used to “start” the production of the desired compounds by a cell factory.

Wei et al. explored the ability of engineered yeast cells to synthesize carnosic acid. Carnosic acid (salvin) is a plant-derived phenolic tricyclic diterpene with anti-inflammatory, anticancer, and antidiabetic activity. Since traditional plant extraction usually results in low yields (because diterpenoids are often present in low quantities), the authors constructed a biosynthetic pathway in *S. cerevisiae* to generate carnosic acid efficiently. Based on the synthesis of the carnosic acid precursor, miltiradiene, a carnosic acid production strain was developed by integrating the genes encoding cytochrome P450 enzymes and cytochrome P450 reductase. In addition, the co-expression of CYP76AH1 and SmCPR t28SpCytb5 fusion proteins, as well as the overexpression of various catalases to detoxify hydrogen peroxide, resulted in a higher concentration of carnosic acid. This study opens up perspectives for improved microbial biosynthesis of other diterpenoids.

Ruger-Herreros et al. delivered a study that investigates how the production of carotenoids is regulated under various stress conditions in the filamentous fungus *Fusarium fujikuroi*—both in cells deficient for the master regulator CarS and cells complemented with the CarS gene. The authors tested four stresses—heat, nitrogen starvation, light, and oxygen stress—to find that the CarS protein is not involved in the same manner in the four responses, and additionally show that regulation through heat shock goes along with differentially spliced CarS mRNA transcripts. *F. fujikuroi* is a model organism for secondary metabolite production and understanding how secondary metabolism is naturally regulated is crucial for designing effective cell factories in the future. As an example, this work revealed that the CarS deletion variants showed increased and continuous production levels of carotenoids under light stress, as the normal adaptation to this stress is eliminated. This would be a potential way to increase the production of various industrially-relevant carotenoids, important antioxidant ingredients and colorants for food and cosmetics.

Zhao et al. reported the engineering of the oleaginous yeast *Rhodosporidium toruloides*—a species that is naturally efficient at producing and accumulating neutral lipids—to form diacylglycerols and free fatty acids. More specifically, the authors focused on genes involved in the interconversion between diacylglycerols and triacylglycerols. RNA interference was employed to downregulate the expression of DGA1, LRO1, and ARE1, and the overexpression of the native TGL5 fused with LDP1 was performed to increase the diacylglycerol and free fatty acid output. This work is important in that it sheds light on new methods to produce lipids, which are important primary commodities in the food, chemicals, and biofuel industries.

Besides using fungi as factories for chemicals, their functional diversity, for instance in enzymes that have evolved to interact with the environment, can be harnessed for environmental pollution monitoring. Soares et al. provided a comprehensive overview of how fungi can be applied as biological whole-cell indicators able to respond to several soil or water toxicants such as metals, organic compounds, and inorganic contaminants. These fungal whole-cell bioassays mostly rely on the natural response of these fungi to the toxicants and can be read out using inhibition of enzymatic activity, mycelium growth, ergosterol content, and bioluminescence intensity as parameters. Additionally, the authors point to strategies for how genetic engineering approaches in yeasts can be harnessed to build advanced biosensors.

This Research Topic showcases different bioengineering strategies across fungal species—from laboratory *S. cerevisiae* strains, to wine yeast, to filamentous fungi such as *Fusarium*—highlighting the increasing diversity of fungal organisms that are becoming benchmarks of what we know today as living cell factories. A steady flow of research on genetic engineering of an increasing variety of fungi will enable future applications of these organisms as cell factories, which in turn may prove valuable for more sustainable societies.

